# Kinetic analysis and test-retest variability of the radioligand [^11^C](R)-PK11195 binding to TSPO in the human brain - a PET study in control subjects

**DOI:** 10.1186/2191-219X-2-15

**Published:** 2012-04-23

**Authors:** Aurelija Jučaite, Zsolt Cselényi, Annie Arvidsson, Gabrielle Åhlberg, Per Julin, Katarina Varnäs, Per Stenkrona, Jan Andersson, Christer Halldin, Lars Farde

**Affiliations:** 1 AstraZeneca Global Clinical Development, Södertälje 151 85, Sweden; 2 PET Centre, Department of Clinical Neuroscience, Karolinska Institutet, Karolinska University Hospital, Stockholm 171 77, Sweden; 3 CNS/Pain iMED, AstraZeneca R&D, Södertälje 151 85, Sweden

**Keywords:** translocator protein, [^11^C](R)-PK11195, positron-emission tomography, peripheral benzodiazepine receptors

## Abstract

**Background:**

Positron-emission tomography and the radioligand [^11^C](R)-PK11195 have been used for the imaging of the translocator protein (TSPO) and applied to map microglia cells in the brain in neuropsychiatric disorders. [^11^C](R)-PK11195 binding has been quantified using reference region approaches, with the reference defined anatomically or using unsupervised or supervised clustering algorithms. Kinetic compartment modelling so far has not been presented. In the present test-retest study, we examine the characteristics of [^11^C](R)-PK11195 binding in detail, using the classical compartment analysis with a metabolite-corrected arterial input function.

**Methods:**

[^11^C](R)-PK11195 binding was examined in six control subjects at two separate occasions, 6 weeks apart. Results of one-tissue and two-tissue compartment models (1TCM, 2TCM) were compared using the Akaike criteria and *F*-statistics. The reproducibility of binding potential (*BP*_ND_) estimates was evaluated by difference in measurements (error in percent) and intraclass correlation coefficients (ICCs).

**Results:**

[^11^C](R)-PK11195 binding could be described by 2TCM which was the preferred model. Measurement error (in percent) indicated good reproducibility in large brain regions (mean error: whole brain 4%, grey matter 5%), but not in smaller subcortical regions (putamen 25%, caudate 55%). The ICC values were moderate to low, highest for the white matter (0.73), whole brain and thalamus (0.57), and cortical grey matter (0.47). Sizeable [^11^C](R)-PK11195 *BP*_ND _could be identified throughout the human brain (range 1.11 to 2.21).

**Conclusions:**

High intra-subject variability of [^11^C](R)-PK11195 binding limits longitudinal monitoring of TSPO changes. The interpretation of [^11^C](R)-PK11195 binding by 2TCM suggests that the presence of specific binding to TSPO cannot be excluded at physiological conditions.

## Background

The translocator protein (TSPO), formerly known as the peripheral benzodiazepine receptor (PBR), is widely expressed in peripheral organs, in white blood cells and in the brain parenchyma, predominantly in glial cells [1,2]. The subcellular localisation of TSPO is primarily at the outer mitochondrial membrane, where the TSPO is part of a multimeric protein complex including the voltage-dependent anion channel protein required for benzodiazepine binding and an adenine nucleotide carrier of yet unknown function [3]. A functional role of TSPO and the entire multimeric protein complex has been implicated in a range of cellular biochemical pathways, such as cholesterol transport and steroidogenesis, membrane biogenesis, immunomodulation and oxidative processes [3].

The first radioligand developed for examination of TSPO binding *in vitro *was [^3 ^H]PK11195 [1-(2-chlorophenyl)-*N*-methyl-*N*-(1-methylpropyl)-3-isoquinoline carboxamide] [4]. [^3 ^H]PK11195 is a highly selective antagonist at the TSPO with a nanomolar affinity in rat (*K*_d _= 1.4 nM) and human brain tissue (*K*_d _= 4.3 to 6.6 nM) *in vitro *[5]. The radioligand [^3 ^H]PK11195 has been used to examine TSPO density in the brain and showed increased binding in brain injury as well as neurodegenerative and neuroinflammatory disorders [6,7].

Subsequent molecular imaging studies using ^11^C-labelled PK11195 have been in line with postmortem findings, i.e. increased TSPO binding has been observed in patients with multiple sclerosis, Alzheimer's disease, Parkinson's disease, epilepsy, encephalitis and other central nervous system (CNS) disorders (see review [8]). Despite a low signal-to-background ratio, [^11^C](R)-PK11195 binding to TSPO has been suggested as a potential biomarker of neuroinflammatory and neurodegenerative processes. A common assumption in studies with [^11^C](R)-PK11195 is that the radioligand has no specific binding in the brain *in vivo *at physiological conditions. However, even with the wide applications of [^11^C](R)-PK11195, a full kinetic compartment analysis has not been published, and the reproducibility of [^11^C](R)-PK11195 binding is limited to linear graphical, spectral cluster analysis and reference region-based methods [9].

The purpose of the present study was to examine [^11^C](R)-PK11195 binding using a full kinetic compartment analysis with a metabolite-corrected arterial input function. Six control subjects were examined twice, on separate days, so as to extend test-retest examination for compartment analysis.

## Methods

The study was approved by the Regional Ethics Committee in Stockholm and the Radiation Protection Committee at the Karolinska University Hospital, Stockholm. Written informed consent was obtained from each subject.

### Subjects

The study group consisted of six subjects, all men, mean age 25.8 ± 3.9 years (range 21 to 31 years). Participants were recruited at the AstraZeneca Clinical Pharmacology Unit, Karolinska University Hospital, Huddinge, Stockholm. They were healthy according to the medical history, clinical examination, and routine laboratory blood and urine tests. No medications were used at the time of the study. No anatomical brain abnormality was detected on magnetic resonance imaging (MRI) in any subject as evaluated by a neuroradiologist at the Karolinska University Hospital.

The molecular imaging examinations were carried out at the PET Centre, Department of Clinical Neuroscience, Karolinska Institutet, Stockholm. All subjects underwent two positron-emission tomography (PET) measurements approximately 6 weeks apart.

### Radiochemistry

[^11^C](R)-PK11195 was prepared by *N*-methylation of the desmethyl precursor at room temperature, with slight modifications of a synthesis [10]. In short, [^11^C]methane was produced in-target via the ^14 ^N(p,α)^11^C reaction on a N_2_-H_2 _gaseous system, in a GEMS PET trace cyclotron. [^11^C]methyl iodide was produced according to previously published methods [11]. [^11^C]CH_3_I was trapped in a solution of a precursor (0.5 mg) and potassium hydroxide (5 mg) in dimethylsulfoxide (300 μL). After the synthesis of [^11^C](R)-PK11195, the radioligand was dissolved in a sterile disodium phosphate-buffered saline solution, pH 7.4, and filtered through a sterile Millex-GV filter (0.22 μm; Millipore AB, Solna, Sweden).

The mean decay-corrected radiochemical yield (*n *= 12) of [^11^C](R)-PK11195 was 9.1%, and the radiochemical purity of the final product was > 98%. The specific radioactivity of the radioligand injected varied between 421 and 2,303 GBq/μmol (11,390 and 62,249 Ci/mmol), corresponding to an injected total mass of 0.05 to 0.23 μg. The radioactivity injected was 302 ± 33 MBq (mean ± standard deviation (SD); 12 measurements).

### MR imaging

The three-dimensional (3D) MR protocol comprised Siemens 3D T1-weighted magnetization-prepared rapid acquisition with gradient echo (MPRAGE) sequences. Images were obtained with a Siemens 1.5 T Magnetom with an eight-channel parallel coil, using a GRAPPA-accelerated protocol with 3D sequences covering the whole brain with isotropic voxel sizes (1.3 × 1.3 × 1.3 mm) [12].

### PET measurement procedure

Each subject was placed recumbent with the head in the PET system. A head fixation system with an individual plaster helmet was used. A cannula was inserted into the left radial artery and another into the right antecubital vein. A sterile phosphate buffer (pH = 7.4) containing radioligand was injected as a bolus over 2 s into the cubital vein. The cannula was then immediately flushed with a 10-mL saline solution. In each individual, the measurement was repeated after approximately 6 weeks.

The PET system used was ECAT Exact HR 47 (Siemens/CTI, Knoxville, TN, USA), with an in-plane and an axial resolution of 3.6 and 4.0 mm, respectively, full width at half maximum (FWHM). The radioactivity in the brain was measured continuously for 60 min using a pre-programmed sequence of 29 frames (20 s × 9, 30 s × 6, 180 s × 5, 300 s × 5). The system was run in 3D mode, and the reconstructed volume was displayed as 47 sections with a centre-to centre distance of 3.125 mm. The images were reconstructed with a Hanning filter (FWHM = 2 mm) in a matrix of 128 × 128 × 47 voxels, yielding a voxel size of 2.03 × 2.03 × 3.125 mm. Attenuation correction was done using transmission scan data obtained for each subject.

### Arterial blood sampling

To obtain the arterial input function, an automated blood sampling system was used during the first 5 min of each PET measurement. Thereafter, arterial blood samples (2 mL) were drawn manually at the midpoint of each frame until the end of measurement [13]. The radioactivity in 1 mL of the manually drawn samples was then immediately measured for 10 s in a well counter cross-calibrated with the PET system. After centrifugation, 0.2 mL plasma was pipetted and plasma radioactivity was measured in a well counter.

### Plasma metabolite analysis of [^11^C](R)-PK11195 using HPLC

The method for the determination of the fraction of radioactivity corresponding to unchanged [^11^C](R)-PK11195 and labelled metabolites during the time of a PET measurement was a modification of a high-performance liquid chromatography (HPLC) method developed for other PET radioligands [14]. Arterial blood samples (2 mL) were obtained at 4, 10, 20, 30, 40 and 50 min after injection of [^11^C](R)-PK11195. After centrifugation at 2,000 × *g *for 2 min, plasma was obtained (0.5 mL) and mixed with acetonitrile (0.7 mL). The mixture was centrifuged at 2,000 × *g *for 2 min, and the supernatant (1 mL) was injected to a HPLC system. The blood (2 mL) and plasma (0.5 mL) were counted in a NaI well counter. The HPLC system used consisted of a Merck-Hitachi D-7000 interface module, a Merck-Hitachi L-7100 pump and a Rheodyne injector (7125 with a 5.0-mL loop) (VWR International, Stockholm, Sweden) equipped with a Waters/-Bondapack-C18 column (300 mm × 7.8 mm, 10-μm particle size; Waters, Milford, MA, USA) and a Merck-Hitachi L-7400 absorbance detector (254 nm) in series with a Packard Radiomatic 150TR radiometric detector (Packard BioScience, Meriden, CT, USA) equipped with a PET flow cell (600-μL cell). The HPLC methods used were acetonitrile (A) and phosphoric acid (0.01 M) (B) as mobile phase at 6.0 mL/min. The program was as follows: 0 to 5 min (A/B) 35/65 to 80/20, 5 to 7.5 min (A/B) 80/20 to 35/65 and 7.5 to 10 min (A/B) 35/65. The radioactive peak having a retention time corresponding to [^11^C](R)-PK11195 was integrated, and its area was expressed as a percentage of the sum of the areas of all detected radioactive peaks.

### Image analysis

#### MR image pre-processing

The T1-weighted MPRAGE images were co-registered to a template atlas, inhomogeneitycorrected, masked and segmented through a fuzzy c-means cluster algorithm into grey matter, white matter and cerebrospinal fluid membership images using the in-house software BMAP/Volstat [12].

The original MR images and segmented grey and white matter images were re-oriented and re-aligned to the anterior-posterior commissural plane, re-sampled and cropped to generate a 256 × 256 × 144 matrix with 1-mm^3 ^voxels.

#### PET image pre-processing

The 4D PET images were integrated over time to obtain 3D PET summation images. The PET images were co-registered to the re-oriented, high-resolution T1-weighted MRI. The summation PET images were used to determine the co-registration parameters (rotation and translation required). The co-registered PET images were re-sliced to a voxel size of 2 × 2 × 2 mm. The spatial processing of MR and PET images was performed in SPM5 (Wellcome Department of Cognitive Neurology, UK).

#### Regions of interest

Regions of interest (ROIs) were processed in two different ways. Anatomically defined ROIs (caudate nucleus, putamen, thalamus and brainstem) were manually delineated on the reoriented T1-weighted MR image using an in-house image analysis software, Human Brain Atlas. Three additional brain tissue ROIs (whole brain, all grey matter and all white matter) were obtained automatically. A whole brain region was created taking the union of all white and grey matter. The additional 'grey matter' and 'white matter' ROIs were derived by the following steps: (1) MR image segmentation into the white and grey matter, (2) creation of a thalamus-brainstem mask using anatomical ROIs, (3) subtraction of the mask in (2) from the segmented regions in (1). These regions represent the grey matter and the white matter without the brainstem-thalamus regions, respectively. The grey matter region was coregistered to summation PET image voxels. The most common radioactivity values were used to create a brain region representing a 'typical grey matter (GM)' ROI.

#### Radioligand brain exposure

To calculate the percentage of [^11^C](R)-PK11195 injected that was present in the brain at the time of maximal radioactivity, the radioactivity concentration in the ROI for the whole brain was multiplied by the estimated average brain volume (1,250 mL), divided by the radioactivity injected and multiplied by 100 to obtain the percentage of injected radioactivity.

#### Regional brain radioactivity

Average radioactivity for the ROI was calculated by pooling the data from the series of brain sections. The radioactivity concentration for each sequential time frame was corrected for the physical decay of carbon-11 and used to obtain the regional time-radioactivity curves (TACs; in kiloBecquerels per cubic centimetre).

### Arterial plasma input function

Arterial blood data from the manual samplings were interpolated to obtain curves with one data point per second up to the end of PET acquisition. The interpolation was performed using a weighted curve fit with a smoothing factor of 0%. Blood time-activity curves were generated by connecting the initial automated blood sampling system curve with the interpolated curve from later manual blood samples. Plasma and blood radioactivity concentrations from manual blood samplings were divided to obtain a plasma/blood ratio curve. The ratio curve was inter- and extrapolated from 0 s up to the end of acquisition by a linear fit. The extended ABSS curve was multiplied with the fitted plasma/blood ratio curve to obtain the plasma curve. Correction for dispersion as well as correction for radioligand metabolism was performed as has been described previously [13]. Individual parent fraction data were fitted using the Hill model [15]. Metabolite correction was performed by multiplying uncorrected plasma curves with parent fraction model curves.

### Quantifications of [^11^C](R)-PK11195 binding

[^11^C](R)-PK11195 binding to TSPO was quantified using kinetic compartment analyses with the metabolite-corrected arterial plasma curve as input function. Two compartment models (one- and two-tissue) were used to fit the model to the TACs. The fractional volume of blood present in the tissue volume and bolus time were estimated for each individual, each occasion, by fitting the whole brain TAC with a two-tissue compartment model.

#### The one-tissue compartment model

The one-tissue compartment model (1TCM) describes one compartment which is a composite of free, non-specifically and specifically bound radioligands [16]. The two rate constants, *K*_1 _and *k*_2_, describe the influx and efflux rates across the blood-brain barrier, respectively [17].

#### The two-tissue compartment model

The two-tissue compartment model (2TCM) has been described in detail in the literature [13,18,19]. The two-tissue compartments are defined as the radioactivity concentration of a nondisplaceable radioligand in the brain (*C*_ND_) and the radioactivity concentration of a radioligand specifically bound to receptors (*C*_B_). In this model, four rate constants (*K*_1_, *k*_2_, *k*_3 _and *k*_4_) explain the time curves for radioligand in the brain. In short, the four rate constants were determined by curve fitting with a weighted non-linear least squares fitting technique with constraint-restricting parameters between 0 and 0.9. Each initial value for *K*_1_, *k*_2_, *k*_3 _and *k*_4 _was 0.5, 0.1, 0.5 and 0.5, respectively. The local minimum of the sum of the squared residuals was determined by iteration.

Regional [^11^C](R)-PK11195 binding was expressed using the concept of the total distribution volume (*V*_T_), which is defined by the following equation, using kinetic constants obtained from 2TCM [16,20,21].

$$V_{\text{T}}=K_{1}/k_{2}\left(1+k_{3}/k_{4}\right)$$

The [^11^C](R)-PK11195 distribution volume was also quantified using a linear graphical analysis for reversible ligand binding [22]. The regional distribution volumes were determined from the slope of the linear plots obtained from 8 to 60 min after intravenous (i.v.) injection using the radioactivity of unchanged [^11^C](R)-PK11195 in arterial plasma as an input function.

Specific [^11^C](R)-PK11195 binding was expressed as binding potential relative to nondisplaceable binding (*BP*_ND_) as follows:

$$BP_{\text{ND}}=f_{\text{ND}}B\max/K_{\text{d}}=k_{3}/k_{4}$$

where *B*_max _is the receptor density, *K*_d _is the affinity, and *f*_ND _is the free fraction of ligand in the nondisplaceable tissue compartment [19]. However, protein binding of [^11^C](R)-PK11195 in plasma was not measured in the study, and *f*_ND _was not included in the calculations.

The fractional volume of blood present in the tissue volume was determined for each individual, each occasion. It was obtained by fitting whole brain TAC with a 2TCM with six parameters (*K*_1_, *k*_2_, *k*_3_, *k*_4_, fractional blood volume (*V*_B_) and time shift between plasma input function and PET TAC (*t*_bolus_)) and parameters *V*_B _and *t*_bolus _subsequently applied for each brain region.

#### The two-tissue compartment model, modified

In addition, data were analysed using a modified 2TCM model which fitted parameters *K*_1_/*k*_2_, *k*_2_, *k*_3 _and *k*_4 _of the model to several TACs simultaneously in a way that the parameter *K*_1_/*k*_2 _was shared between regions. First, all ROIs primarily consisting of the grey matter, including the ROI of the typical GM uptake, were fitted in this way then followed by a fit involving white matter regions and, again, the ROI of the typical GM uptake. The ROI of the typical GM uptake was used in the second fitting session to allow for a coupling of *K*_1_/*k*_2 _between that region and white matter ROIs. However, the parameters obtained for the the typical GM ROI in the first session were retained for final analysis.

### Statistics

The Akaike information criterion [23] and *F*-statistics [13] were used to determine which compartment model provided the best fit to the data. *F*-statistics was one-tailed (right).

The reproducibility of two successive PET measurements was analysed by means of descriptive statistics (mean, range, SD, coefficient of variance (CV), difference in measurement (error in percent) and intra-class correlation coefficient (ICC)). The ICC was calculated from the between-subject and within-subject variations, using the expression:

$$\text{ICC}=(\text{M}{\text{S}}_{\text{BS}}-\text{M}{\text{S}}_{\text{WS}})/\left[\text{M}{\text{S}}_{\text{BS}}+(n-1)\text{M}{\text{S}}_{\text{WS}}\right]$$

where MS_BS _denotes the between-subject mean square; MS_WS_, the within-subject mean square; and *n*, the number of repeated observations (i.e. 2).

The descriptive statistics and computations for test-retest analysis were performed using the STATISTICA system, version 10.0 (StatSoft, Inc., Tulsa, OK, USA). In all analyses, the statistical significance (alpha level) was set at *p *< 0.05.

## Results

**Figure 1:**
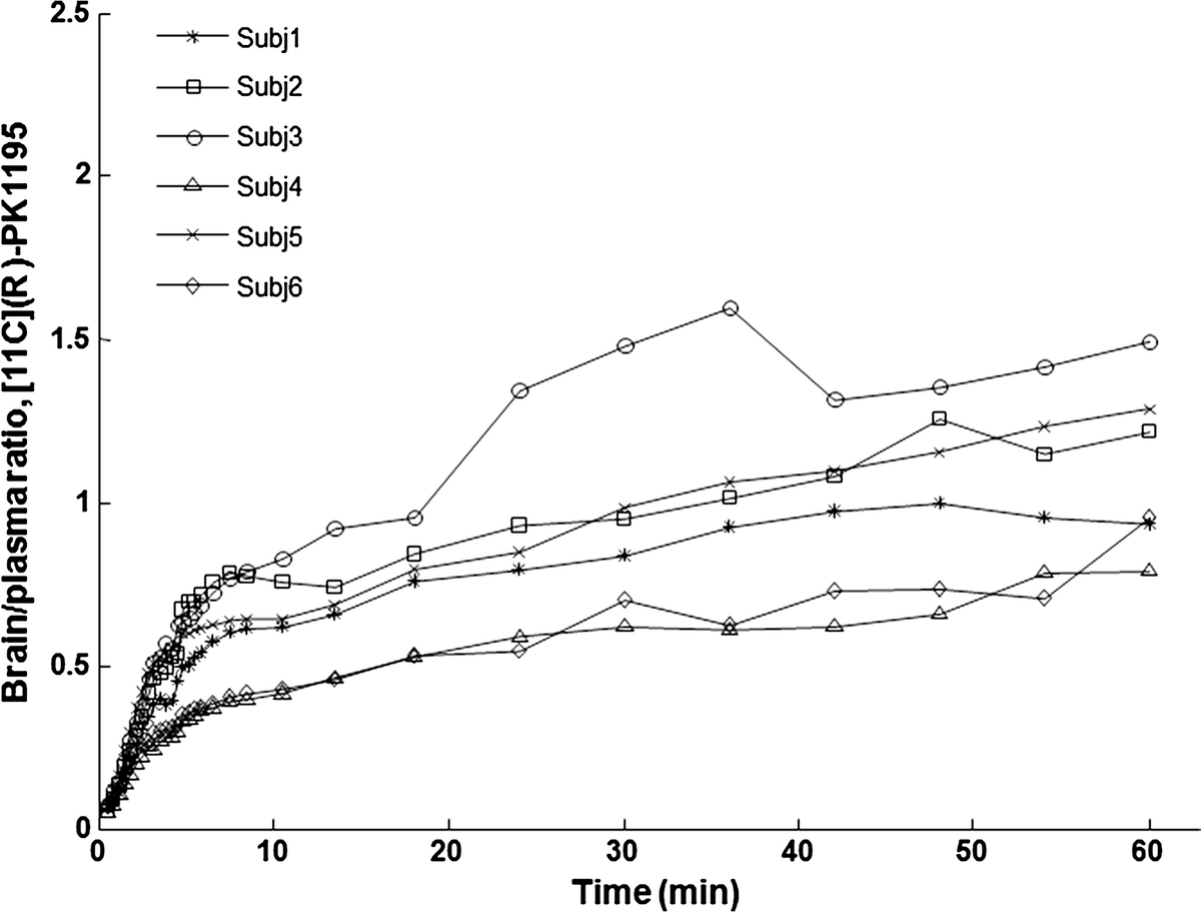
**Ratio of brain concentration of [^11^C](R)-PK11195 to plasma concentration over time**. (Plasma concentration is corrected for metabolites).

After intravenous injection of [^11^C](R)-PK11195, the TACs for total brain radioactivity reached a peak at approximately 1 min after injection. At this time, about 2.5% (range from 1.9% to 3.2%, *n *= 6) of radioactivity injected (ID%) was present in the brain, and mean ID% decreased to about 1.5% to 1.3% during the measurement time from 20 to 60 min. The radioactivity ratio of whole brain to plasma (corrected for metabolites) increased with time and ranged between 0.5 and 2 (*n *= 6) by the end of the PET measurement (Figure 1).

**Figure 2:**
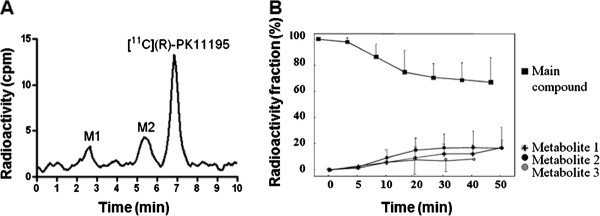
**Metabolism of [^11^C](R)-PK11195**. (**A**) Radiochromatogram from gradient HPLC analysis of human plasma after i.v. injection of [^11^C](R)-PK11195 (peak III). Peaks I and II represent labelled metabolites that are more polar than unchanged [^11^C](R)-PK11195. (**B**) Time course for the fraction (%) of radioactivity in plasma that represents unchanged [^11^C](R)-PK11195 and its metabolites in six healthy human subjects (mean, SD).

**Figure 3:**
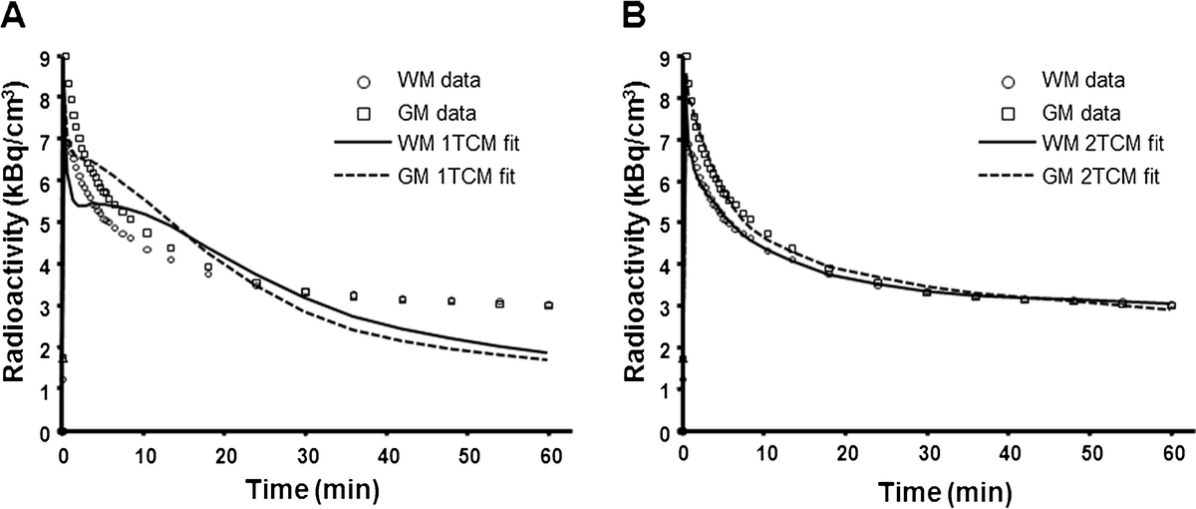
**Experimental values for regional radioactivity in the grey and white matter in one subject**. The corresponding fitted curves are obtained by the 1TCM (**A**) and by the 2TCM (**B**).

HPLC analysis of [^11^C](R)-PK11195 metabolites in plasma identified the parent compound and radioactive metabolites (Figure 2A, sample at 20 min after injection). The metabolites were more polar than the parent compound. The unchanged fraction of [^11^C](R)-PK11195 was 70 ± 1.5% (range 60% to 77%) by the end of PET measurement time (Figure 2B). For all brain regions, the 1TCM and the 2TCM were fitted to experimental data using the metabolite-corrected arterial plasma curve as input function (Figure 3A, B).

**Figure 4:**
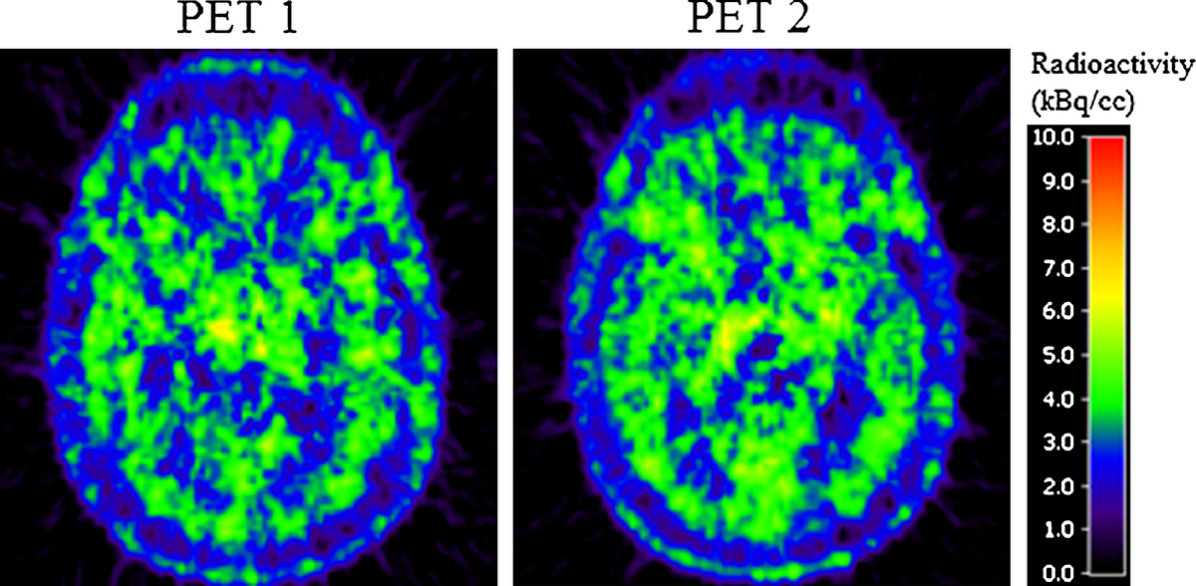
**PK11195 summation images, test-retest (9- to 60-min imaging interval; normalized image intensity for injected radioactivity)**.

The single rate constants obtained by the 1TCM and 2TCM during repeated PET measurements are given in Tables 1 and 2. The ratio of *K*_1 _to *k*_2 _was obtained by the 2TCM. The comparison of models showed that the 2TCM was statistically preferred to describe the time-activity curves of [^11^C](R)-PK11195 in all brain regions and in all subjects. The fractional cerebral blood volume across all occasions ranged from 4.4% to 7% (6 ± 0.009%, mean ± SD). In addition, the fitting procedure was carried out with the modified 2TCM-2 using a shared *K*_1_/*k*_2_. Kinetic constant values were similar to the ones obtained by classical 2TCM, and there was no significant improvement in statistics compared to 2TCM data (Akaike criteria) (see Table 3 and Tables 1 and 2 for comparison). Numerical values for specific binding (*BP*_ND _= *k*_3_/*k*_4_) throughout the brain regions varied twofold (Table 4, Figure 4).

**Table 1:** Comparison of kinetic constants in 1TCM and 2TCM for [^11^C](R)-PK11195 binding in the grey matter

Subject	Test (1); retest (2)	Model	*K*_1 _(mL/cc/min)	*k*_2 _(min^-1^)	*k*_3 _(min^-1^)	*k*_4 _(min^-1^)	*K*_1_*/k*_2 _	*k*_3_*/k*_4 _(*BP*_ND_)	AIC	*F*-statistics (1TCM/2TCM; *p *value)
1	1	1TCM	0.074	0.116			0.64		239	
		2TCM	0.094	0.264	0.076	0.067	0.36	1.13	171	3 × 10^-14^
	2	1TCM	0.056	0.099			0.57		264	
		2TCM	0.073	0.234	0.059	0.044	0.31	1.33	197	4 × 10^-14^
2	1	1TCM	0.054	0.096			0.55		253	
		2TCM	0.072	0.252	0.058	0.026	0.28	1.86	173	1 × 10^-16^
	2	1TCM	0.057	0.118			0.48		274	
		2TCM	0.077	0.287	0.059	0.041	0.27	1.44	211	2 × 10^-13^
3	1	1TCM	0.062	0.086			0.72		264	
		2TCM	0.082	0.220	0.061	0.033	0.39	1.55	181	< 1 × 10^-323^
	2	1TCM	0.094	0.111			0.84		286	
		2TCM	0.129	0.318	0.111	0.076	0.41	1.47	210	9 × 10^-16^
4	1	1TCM	0.037	0.087			0.41		236	
		2TCM	0.048	0.220	0.055	0.038	0.22	1.45	179	3 × 10^-12^
	2	1TCM	0.035	0.086			0.41		236	
		2TCM	0.049	0.246	0.062	0.035	0.20	1.80	178	2 × 10^-12^
5	1	1TCM	0.062	0.111			0.56		283	
		2TCM	0.082	0.245	0.040	0.023	0.33	1.75	184	< 1 × 10^-323^
	2	1TCM	0.044	0.108			0.41		288	
		2TCM	0.067	0.340	0.068	0.032	0.20	2.08	204	< 1 × 10^-323^
6	1	1TCM	0.042	0.106			0.39		267	
		2TCM	0.054	0.221	0.038	0.029	0.24	1.27	197	1 × 10^-4^
	2	1TCM	0.045	0.091			0.49		272	
		2TCM	0.060	0.239	0.065	0.045	0.25	1.44	231	3 × 10^-9^
Mean	1	1TCM	0.055(0.014/25)	0.100(0.012/12)			0.54(0.13/23)	Mean	1	1TCM
(SD/CV)		2TCM	0.072(0.018/25)	0.237(0.019/8)	0.055(0.014/25)	0.029(0.011/38)	0.30(0.06/21)	1.50(0.27/18)	(SD/CV)	
	2	1TCM	0.055(0.021/18)	0.102(0.012/12)			0.53(0.16/31)			
		2TCM	0.076 (0.028/37)	0.277(0.045/16)	0.071(0.02/28)	0.046(0.016/34)	0.27(0.08/29)	1.59(0.29/18)		

Data from the first PET measurement used for all subjects. 1TCM, one-tissue compartment model; 2TCM, two-tissue compartment model; SD, standard deviation; CV, coefficient of variation; AIC, Akaike information criteria

**Table 2:** Comparison of kinetic constants in 1TCM and 2TCM for [^11^C](R)-PK11195 binding in the white matter

Subject	Test (1); retest (2)	Model	*K*_1 _(mL/cc/min)	*k*_2 _(min^-1^)	*k*_3 _(min^-1^)	*k*_4 _(min^-1^)	*K*_1_*/k*_2_	*k*_3_*/k*_4 _(*BP*_ND_)	AIC	*F*-statistics (1TCM/2TCM; *p *value)
1	1	1TCM	0.055	0.086			0.64		228	
		2TCM	0.067	0.165	0.037	0.030	0.41	1.21	180	2 × 10^-10^
	2	1TCM	0.042	0.071			0.59		250	
		2TCM	0.051	0.144	0.033	0.022	0.35	1.49	198	3 × 10^-11^
2	1	1TCM	0.044	0.071			0.61		244	
		2TCM	0.056	0.176	0.043	0.019	0.32	2.20	177	4 × 10^-14^
	2	1TCM	0.046	0.089			0.52		267	
		2TCM	0.060	0.206	0.042	0.025	0.29	1.65	208	1 × 10^-12^
3	1	1TCM	0.049	0.066			0.74		250	
		2TCM	0.061	0.154	0.043	0.026	0.40	1.67	177	3 × 10^-15^
	2	1TCM	0.075	0.085			0.88		278	
		2TCM	0.097	0.209	0.061	0.045	0.61	1.36	212	7 × 10^-14^
4	1	1TCM	0.027	0.066			0.41		223	
		2TCM	0.032	0.124	0.024	0.019	0.26	2.03	192	3 × 10^-7^
	2	1TCM	0.027	0.066			0.41		239	
		2TCM	0.035	0.164	0.041	0.020	0.21	2.03	176	2 × 10^-13^
5	1	1TCM	0.048	0.081			0.59		274	
		2TCM	0.061	0.184	0.034	0.014	0.33	2.41	201	3 × 10^-15^
	2	1TCM	0.032	0.078			0.41		273	
		2TCM	0.045	0.223	0.046	0.019	0.20	2.50	208	1 × 10^-13^
6	1	1TCM	0.030	0.072			0.41		255	
		2TCM	0.035	0.138	0.024	0.012	0.25	1.91	213	2 × 10^-9^
	2	1TCM	0.033	0.064			0.52		246	
		2TCM	0.039	0.126	0.028	0.018	0.31	1.62	175	8 × 10^-15^

Mean	1	1TCM	0.042(0.011/26)	0.074(0.008/11)			0.57(0.13/23)			
(SD/CV)		2TCM	0.052(0.015/29)	0.157(0.02/13)	0.034(0.009/26)	0.020(0.007/35)	0.33(0.07/20)	1.93(0.48/22)		
	2	1TCM	0.043(0.017/39)	0.076(0.010/13)			0.56(0.18/32)			
		2TCM	0.055(0.023/42)	0.179(0.040/22)	0.042(0.011/26)	0.025(0.010/40)	0.31(0.10/32)	1.78(0.42/24)		

Data from the first PET measurement used for all subjects. 1TCM, one-tissue compartment model; 2TCM, two-tissue compartment model; SD, standard deviation; CV, coefficient of variation; AIC, Akaike information criteria

**Table 3:** Kinetic constants in 2TCM with shared *K*_1_/*k*_2 _values for [^11^C](R)-PK11195 binding in 6 healthy subjects

Subject	*K*_1 _(mL/cc/min)	*k*_2 _(min^-1^)	*k*_3 _(min^-1^)	*k*_4 _(min^-1^)	*K*_1_*/k*_2_	*k*_3_*/k*_4 _(*BP*_ND_)	AIC
Cortical grey matter							

1	0.092	0.236	0.055	0.054	0.39	1.02	170
2	0.070	0.228	0.046	0.025	0.31	1.82	173
3	0.089	0.307	0.124	0.061	0.29	2.01	195
4	0.046	0.185	0.035	0.026	0.25	1.36	180
5	0.080	0.235	0.036	0.020	0.34	1.80	183
6	0.050	0.180	0.021	0.014	0.28	1.47	204
Mean	0.071	0.228	0.053	0.033	0.31	1.58	
(SD/CV)	(0.019/27)	(0.046/20)	(0.037/69)	(0.019/57)	(0.05/16)	(0.36/23)	

White matter							

1	0.067	0.169	0.040	0.033	0.40	1.02	178
2	0.056	0.180	0.045	0.021	0.31	2.18	176
3	0.062	0.159	0.046	0.027	0.39	1.68	176
4	0.032	0.129	0.026	0.013	0.25	1.97	190
5	0.061	0.182	0.033	0.013	0.33	2.46	200
6	0.036	0.138	0.024	0.012	0.26	1.97	212
Mean	0.052	0.159	0.036	0.020	0.32	1.88	
(SD/CV)	(0.015/29)	(0.022/14)	(0.009/25)	(0.009/45)	(0.06/19)	(0.49/26)	

Data from the first PET measurement only is presented for simplification purposes. SD, standard deviation; CV, coefficient of variation; AIC, Akaike information criteria

**Table 4:** Test-retest characteristics of [^11^C](R)-PK11195 binding to the TSPO in healthy subjects

Brain region			*BP*_ND _(*k*_3_*/k*_4_)			Error range (%)	Error % mean(SD)	ICC
				
	Test	CV (%)	Retest	CV (%)	Mean			
Caudate	1.37(0.55)	40	1.92(0.87)	45	1.65	-155 to 30	-55(80)	0.01
Caudate (R)	1.33(0.68)	51	2.21(1.19)	54	1.77	-288 to12	-96(126)	0.01
Caudate (L)	1.52(0.43)	28	1.84(0.93)	51	1.68	-133 to 42	-27(66)	-0.05
Putamen	1.20(0.28)	24	1.50(0.69)	46	1.38	-138 to 21	-25(63)	0.09
Putamen (R)	1.27(0.48)	37	1.56(0.73)	47	1.41	-145 to 34	-32(72)	-0.01
Putamen (L)	1.28(0.31)	24	1.60(0.67)	42	1.44	-145 to 23	-30(59)	0.02
Thalamus	1.19(0.28)	23	1.53(0.45)	30	1.36	-82 to 9	-29(33)	0.57
Thalamus (R)	1.34(0.43)	32	1.86(0.56)	30	1.60	-136 to 17	-46(50)	0.16
Thalamus (L)	1.11(0.25)	23	1.42(0.59)	42	1.26	-136 to 39	-32(64)	0.17
Brainstem	2.00(0.35)	17	2.18(0.33)	15	2.09	-45 to 26	-13(31)	-0.6
Cerebellum	1.29(0.19)	15	1.37(0.24)	17	1.33	-21 to 32	-8(20)	0.07
White matter	1.90(0.42)	22	1.78(0.42)	24	1.84	-25 to 21	-8(18)	0.73
Grey matter, cortex	1.50(0.27)	18	1.59(0.29)	18	1.55	-24 to 25	5(18)	0.47
Whole brain	1.65(0.33)	20	1.68(0.35)	21	1.67	-25 to 22	-4(19)	0.57

Binding potential, *BP*_ND _*= k*_3_*/k*_4 _from 2TCM; CV, coefficient of variance; ICC, intra-class correlations

### Test-retest analysis

**Figure 5:**
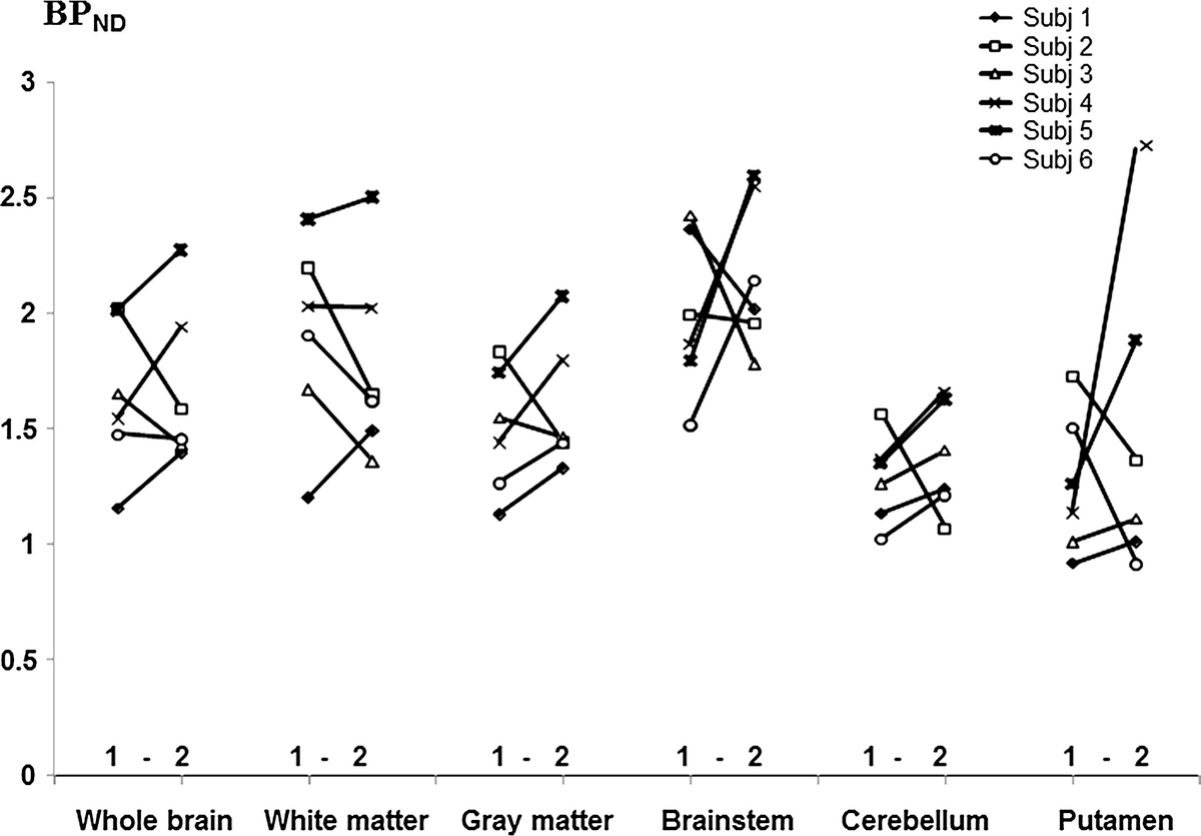
**Binding potentials (*BP*_ND_, *k*_3_/*k*_4_) obtained at repeated PET measurements in six control subjects**.

The test-retest variability for *BP*_ND _(*k*_3_/*k*_4_) varied between brain regions. The mean difference between two PET measurements was lowest in the cortical grey matter (5%), white matter (-4%), cerebellum and white matter (-8%) regions and highest in the dorsal striatum and thalamus (-55% and -29%, respectively) (Table 4, individual data in Figure 5). The ICC values for [^11^C](R)-PK11195 were fair to moderate in the thalamus, grey matter, white matter and whole brain regions, but were of low reproducibility in the striatum, brainstem and cerebellum.

The *V*_T _analysis has been widely used for quantification of [^11^C](R)-PK11195 binding. Test-retest analysis of *V*_T _showed lower error compared to the *BP*_ND _(*k*_3_/*k*_4_) data, though *V*_T _values (for 2TCM) also tended to be higher in the striatum than in the cortex or whole brain (Table 5).

**Table 5:** The total distribution volume obtained by the 2TCM and linear graphical methods

Brain region	*V*_T (2TCM)_	Error range (%)	Error (%) mean(SD)	*V*_T Logan_	Error range (%)	Error (%) mean(SD)
Caudate	0.88(0.13) to 1.06 (0.24)	-35 to 62	25(36)	0.64(0.16) to 0.59(0.19)	-37 to 25	-5(23)
Putamen	0.83(0.21) to 0.93(0.19)	-32 to 78	17(38)	0.70(0.15) to 0.73(0.26)	-25 to 42	3(25)
Thalamus	0.90(0.27) to 0.80 (0.23)	-47 to 40	-6(29)	0.80(0.24) to 0.76(0.25)	-26 to 27	-3(20)
Brainstem	0.86(0.18) to 0.74 (0.16)	-29 to -1	-13(10)	0.74(0.21) to 0.67(0.19)	-24 to 12	-8(12)
Cerebellum	0.74(0.18) to 0.72 (0.16)	-32 to 24	-1(18)	0.72(0.18) to 0.68(0.18)	-29 to 20	-4(18)
Grey matter	0.76(0.18) to 0.70 (0.17)	-36 to 15	-6(17)	0.69(0.17) to 0.65(0.18)	-28 to 17	-4(16)
White matter	0.99(0.17) to 0.88 (0.19)	-42 to 28	-9(25)	0.69(0.18) to 0.65(0.20)	-32 to 19	-4(19)
Whole brain	0.82(0.18) to 0.75 (0.16)	-38 to 21	-7(20)	0.69(0.17) to 0.65(0.19)	-29 to 17	-4(16)

Values presented as mean(SD). Test-retest values are presented. *V*_T (2TCM) _is the distribution volume of two-tissue compartment model, *K*_1_/*k*_2 _(1 + *k*_3_/*k*_4_). *V*_T Logan _is the distribution volume obtained using the arterial input curve and linear graphical analysis. SD, standard deviation

## Discussion

Several CNS disorders are accompanied by the activation of microglia which is thought to be a part of the disease pathophysiology. In an era of emerging new therapies aiming to modify disease progression, it is critical to have a biomarker that enables the monitoring of disease course and effects of therapeutic interventions at the cellular level. Molecular imaging using radioligand binding to markers of microglia has potential to serve this purpose. Ideally, the imaging signal has to be highly reproducible, sensitive to changes, and the underlying biology has to be well understood. All these features have been in focus in the development of the reference radioligand [^11^C](R)-PK11195 and a subsequent series of radioligands binding to the TSPO.

In the present study, a detailed analysis of [^11^C](R)-PK11195 binding using arterial input function and full kinetic compartment modelling was performed. The two-tissue compartment model was the statistically preferred model, and the *BP*_ND _values were of the same order as seen for commonly used radioligands binding to receptors or transporters. The study results were in line with the *BP*_ND _values presented in by the initial kinetic compartment analysis [24] and clinical studies [25].

The present study included a preliminary analysis of reliability of the quantitative analysis for [^11^C](R)-PK11195 binding potential, *BP*_ND _(*k*_3_/*k*_4_), using a test-retest approach. The mean difference between test and retest was lowest in large regions such as the whole brain, cortical grey matter and white matter, and was high in the smaller brain regions. These reproducibility values are acceptable for studies where analysis of [^11^C](R)-PK11195 binding is limited to larger brain regions, however, is insufficient for quantitative analysis in small regions. In addition, the twofold inter-individual variability in [^11^C](R)-PK11195 *BP*_ND _in this limited sample of six subjects indicates that studies with group comparisons may require large sample size. Another important factor that may contribute to the variability of radioligand binding to TSPO is the presence of several binding sites with different affinities dependent on single nucleotide polymorphism of TSPO, at Ala147Thr [26]. However, high inter-individual variability in [^11^C](R)-PK11195 binding cannot be explained by TSPO SNP variants, as it has been demonstrated that this is the only radioligand not sensitive to TSPO genotype [27].

The [^11^C](R)-PK11195 binding was homogenously distributed in the human brain (Tables 1 and 2, Figure 4, [28]). Following the advent of [^11^C](R)-PK11195, a common assumption has been that the constitutive expression of TSPO in the brain is low or negligible [9,29]. This assumption has paved the way for straightforward analyses where higher binding in a certain brain region is viewed as 'microglia activation' contrasting to a background of non-specific binding. More recently, compartment analysis of [^11^C](R)-PK11195 binding applied in clinical studies has consistently demonstrated a binding compartment also in control subjects [30,31]. However, the nature of this compartment has not been discussed in detail.

In early studies using [^3 ^H]-labelled PK11195, the TSPO density (*B*_max_) in autopsies of normal human brain has been quantified in detail by autoradiography [1]. The TSPO binding sites were shown to be widespread and with high density in several subcortical nuclei, with more moderate binding in the cortex and dorsal striatum. Subsequent studies have consistently confirmed the initial findings, i.e. TSPO density in the grey matter in the range of 684 to 923 fmol/mg protein [8,28,32]. Assuming a 10% protein content in the brain, the corresponding TSPO concentration in intact neural tissue should be 60 to 90 pmol/g. This TSPO concentration is of the same order as for several neuroreceptors, e.g. the dopamine D2/3 receptor density in the striatum (*B*_max _15 pmol/g, [26]) or the 5HT_1A _receptors in the neocortex (up to 120 pmol/g, [33]). For these G-protein-coupled receptors, it has been possible to develop suitable radioligands for molecular imaging. Thus, the TSPO density in the human brain demonstrated *in vitro *conditions should be large enough for quantitative imaging with radioligands having sufficient affinity to TSPO. The affinity of [^11^C](R)PK11195 for TSPO is indeed in the nanomolar range which is comparable to successful neuroreceptor ligands such as [^11^C]raclopride or [^11^C]WAY 100635 (approximately 1 to 3 nM, [13,34]).

The assumption that non-specific [^11^C](R)-PK11195 binding constitutes a major proportion of the signal was based on early *in vivo *studies in baboons [35]. An indirect quantitative approach was used, subtracting TSPO binding from baseline data and suggesting that the fraction of non-specifically bound [^11^C](R)-PK11195 in the grey matter is about 60%. For new radioligands such as [^11^C]DAA1106, specific binding constitutes 80% to 90% of the total binding in the monkey brain [36]. Interestingly, unlabelled PK11195 has been shown to displace about 70% of the total [^11^C]DAA1106 binding [36], and PBR28 could displace [^11^C](R)-PK11195 binding up to 40% to 60% [37], thus, providing indirect support for the presence of sizeable specific TSPO binding.

Quantitative analysis of radioligand binding may be influenced by a number of conditions, including formation of radioactive metabolites that may enter the brain. The present study confirms that the metabolism of [^11^C](R)-PK11195 is not extensive [38] and metabolites less polar than the parent compound could not be identified. Thus, there is no support for the interpretation that a metabolite could cross the BBB and represent the compartment for specific binding identified by the 2TCM.

The 2TCM analysis provided values for individual rate constants, which were in line with the initial results of the kinetic compartment modelling of [^11^C](R)-PK11195 [24]. The influx rate constant *K*_1 _(0.05 to 0.09) was low when compared to *K*_1 _values obtained for other radioligands in the same experimental setting and using the same analytical approach (e.g. 0.16 for [^11^C]raclopride, [39]; 0.42 for [^11^C]FLB-457, [40]). The lipophilicity of [^11^C](R)PK11195 (log *P *= 1.4 to 3.4, [38,41]) is in the range commonly perceived as ideal for a suitable PET ligand (1 to 3). Thus, the slow influx has to be explained by other conditions. Theoretically, one of the reasons for the slower influx rate and lower brain uptake of [^11^C](R)-PK11195 could be its binding to transporters at the blood-brain barrier. However, it has been shown that [^11^C](R)-PK11195 is not a substrate for any of the three major ABC transporters (p-glycoprotein-ABCB1 and ABCC1, ABCG2) [37].

Most radioligands and drug molecules bind to plasma albumin. It has been shown that drug dissociation from albumin is very rapid [42]. Indeed, for [^11^C]raclopride, having a plasma protein binding of 96%, the first pass extraction is about 20%, indicating sizeable dissociation during the short capillary transit time [13]. Interestingly, it has been shown that [^3 ^H]PK11195 binds with high affinity to the alfa1-acid glycoprotein (AGP) and to a lesser extent to human serum albumin (HSA) [43]. The observation that *K*_1 _for [^11^C](R)-PK11195 is lower than that for other radioligands can at least partly be explained by a slower dissociation rate from AGP than from HSA. However, a limitation of the present study was that radioligand protein binding was not determined. If confirmed, this proposed interpretation may have implications for clinical studies on inflammatory disorders known to change the rather stable levels of AGP at physiological conditions [44]. In such conditions, it cannot be excluded that *K*1 may show considerable inter-individual variability.

## Conclusions

The reproducibility of [^11^C](R)-PK11195 binding in large brain regions was acceptable for applied clinical studies. However, the study showed that the use of [^11^C](R)-PK11195 as a biomarker in clinical trial is limited when detailed mapping of TSPO changes in smaller brain regions is needed.

The [^11^C](R)-PK11195 binding to TSPO could be best described by the two-tissue compartment model. The sizeable [^11^C](R)-PK11195 binding demonstrated in the present study is consistent with the literature reporting abundant TSPO binding sites in the human brain post-mortem in control subjects. On the basis of these findings, it cannot be excluded that [^11^C](R)-PK11195 binding at physiological conditions to some degree represents specific binding to TSPO.

## Abbreviations

1TCM: one-tissue compartment model; 2TCM: two-tissue compartment model; 3D: three-dimensional; AGP: acid glycoprotein; *BP*_ND_: binding potential nondisplaceable; CNS: central nervous system; CV: coefficient of variance; FWHM: full width at half maximum; HPLC: high-performance liquid chromatography; HSA: human serum albumin; ICCs: intra-class correlation coefficients; MRI: magnetic resonance imaging; PET: positron-emission tomography; PBR: peripheral benzodiazepine receptor; ROI: region of interest; SD: standard deviation; TACs: time-radioactivity curves; TSPO: translocator protein.

## Competing interests

This work was funded by AstraZeneca Pharmaceuticals. Six of the authors are employees at AstraZeneca Pharmaceuticals. AstraZeneca Pharmaceuticals covers the article-processing charge.

## Authors' contributions

AJ participated in the study design, performed the statistical analysis and drafted the manuscript. ZC performed the MR and PET image analyses and statistical analysis and contributed to the data interpretation and method description. AA conceived the study and participated in the study design and result interpretation. GÅ conceived the study and participated in the study design and interpretation. KV participated in the PET image analysis and method description. PJ designed the MR protocol and performed the MR analysis. PS participated in the study design and carried out the PET examinations. JA performed the radiochemistry work and participated in the data interpretation and description. CH participated in the study design, coordination and interpretation of study results. LF conceived the study and participated in the interpretation of results and manuscript writing. All authors read and approved the final manuscript.
